# Deficit Alternate Drip Irrigation Increased Root-Soil-Plant Interaction, Tomato Yield, and Quality

**DOI:** 10.3390/ijerph17030781

**Published:** 2020-01-27

**Authors:** Jingwei Wang, Yuan Li, Wenquan Niu

**Affiliations:** 1 College of Resources and Environment, Shanxi University of Finance and Economics, Taiyuan 030006, Shanxi, China; 2 Institute of Soil and Water Conservation, Northwest A&F University, Yangling 712100, Shaanxi, China; liy681@nenu.edu.cn; 3 Institute of Soil and Water Conservation, CAS & MWR, Yangling 712100, Shaanxi, China

**Keywords:** alternate drip irrigation, field capacity thresholds, tomato, yield, quality

## Abstract

To determine the soil mechanism in root-zone caused by water saving and the production response to alternate drip irrigation (ADI), the present study investigated the effects of deficit ADI on tomato growth using the conventional surface drip irrigation (CDI) as a control. The interactions among the experimental treatments on root index, photosynthetic efficiency, biomass accumulation, yield, fruit quality and irrigation water use efficiency (*IWUE*) were assessed and the inner mechanism of root-soil effecting on tomato growth, photosynthate distribution, yield and quality was discussed. ADI significantly enhanced root-soil interaction, promoted soil nitrogen and phosphorus absorption by tomato and tomato growth. However, different soil moisture deficits significantly affected tomato photosynthate accumulation and distribution, as well as fruit quality. With irrigation amount of 50% field capacity (*F*), ADI significantly increased soluble sugar, total soluble solid and lycopene by 38.08%, 19.48% and 30.05%, respectively, compared to those of CDI, but decreased irrigation amounts by 29.86% in comparison with the CDI one. ADI of 70% *F* could significantly distribute more photosynthate to fruits, thus enhanced tomato yields by 24.6% and improved *IWUE* by 17.05% compared to that of CDI. In addition, ADI of 70% *F* improved tomato fruits quality, and in particular organic acid was decreased by 43.75% and sugar-acid ratio was increased by 97% compared to CDI. However, ADI of 60% *F* distributed more photosynthate to plant, showing no significant difference of yields in comparison with CDI and ADI of 70% *F*, but a higher *IWUE* by 19.54% than that of CDI. ADI of 60% *F* significantly enhanced soluble sugar, total soluble solid, soluble protein, lycopene and sugar-acid ratio in tomato fruits by 2.06, 1.26, 1.61, 1.4 and 3.2 times respectively compared to CDI. Therefore, ADI of 60% or 70% *F* can be overall recommended for tomato production in a greenhouse, plant growth, fruit yield and quality, and *IWUE*.

## 1. Introduction

Tomato (Solanum lycopersicum) is one of the most popular vegetable loved by people and has the largest cultivating area in the word [1]. Tomato planting acreage and production have been still expanding year by year with the increasing demand from consumers [2]. Tomato needs plenty of water to grow [3], thus water scarcity is the major limiting factor to its growing, especially in arid and semi-arid areas. However, drip irrigation (DI) is reportedly an effective and efficient solution for the contradiction between water demand and water scarcity of agricultural production in arid and semi-arid areas [1]. The effects of different DI methods, irrigation amounts, and water-fertilizer coupling or integration on tomato growth, *IWUE*, yield and fruit quality have been extensively studied in recent years [4,5], but few researchers have studies on the responses of the interconnection among tomato plant growth, photosynthate distribution and fruit quality to different DI methods and irrigation amount.

Alternate drip irrigation (ADI) is an effective DI technique [6]. It can improve crop photosynthesis [7], crop yield and fruit quality [8]; increase *IWUE* by regulating leaf photosynthesis and stomatal conductance [6]; and enhance fertilizer utilization efficiency through reducing soil nitrogen emission [9]. In root-zone, soil and crop root are closely linked forming a dynamically and constantly interacting system, which influences soil material flow and energy exchange between soil and crop and further acts on crop growth [10]. Frequently dry-wet alternate cycles are created in root-zone soil during a short period under ADI conditions, and this would change environmental factors, such as soil water, temperature, and microorganisms [11]. These changes inevitably affect roots growth [12], soil nutrient cycling [13], nutrients absorption by crop [14], photosynthate distribution [15], and crop growth process [16]. As a result, the regulation on the interaction of “root-soil-plant” by dry-wet alternate cycling in root-zone may be the internal mechanism of ADI to save water and increase crop yields. The current studies focus on the effects of soil water and fertilizer on tomato yield and *IWUE* under ADI conditions [17]; however, there is less focus on the interaction of “root-soil-plant” and the mechanism of root-zone soil environment changes affecting on tomato plants growth, photosynthate distribution, and fruit yield and quality. Deficit irrigation is also an effective irrigation technique with high *IWUE*. It can affect tomato biomass accumulation [18], increase tomato early yields and nitrogen absorption by fruit [19], and improve tomato fruit quality and *IWUE* [20,21]; nevertheless, the responses of the inner interaction among these previous indicators to deficit irrigation were less studied.

A further assumption is that the deficit ADI could regulate the interaction of “root- soil-plant” and distributed more photosynthate for fruits to improve yields, fruits quality and irrigation *IWUE*. However, the response of “root-soil-plant” and tomato photosynthate distribution to deficit ADI has not been well addressed. In this study, the effects of deficit ADI on tomato plant growth, photosynthate distribution, *IWUE*, yield and quality were investigated. And, the interactions of “root-soil-plant” were deeply analyzed and discussed. This information would contribute to a better understanding on the inner mechanism of deficit ADI, provide the reference for improving the theoretical basis of drip irrigation, and improve irrigation management level in agriculture, etc.

## 2. Materials and Methods

### 2.1. Experimental Field and Experimental Design

The field experiment was carried out in a plastic greenhouse in Yangling District, Shaanxi Province, China, from October 2014 to May 2015. The soil (0–40 cm) contained 25.4% gravel (2–0.02 mm), 44.1% silt (0.02–0.002 mm), and 30.5% clay particles (<0.002 mm), and it had a bulk density in 1.35 g·cm^−3^, a water capacity with 31.54% (water mass content), and a soil porosity of 49.38%.

The greenhouse was 108 m long (from east to west) and 8 m wide (from south to north) and tomato cultivar “Haidi” (widely cultivated locally) was chosen for the trial. In greenhouse, the planting plots were created by east-west direction, in double ridges per plot. Each plot had an area of 3.6 m^2^ with 6.0 m in length, 0.6 m in ridge width, 0.2 m in height, and 0.3 m in furrow width. There were thirty-four tomato plants in each plot with a 0.35 m spacing between two rows.

The experiment had four treatments: a conventional surface drip irrigation with plastic film treatment (CDI) was set as the control (CK) with a drip irrigation pipe located in the middle of the two tomato rows and the lower and upper limits of irrigation set at 70% and 75% of the field capacity (*F*), respectively; three ADI treatments were set up with the lower limits of irrigation at 50% (A50, 60% (A60), and 70% (A70) *F* and the upper limits at 55%, 65%, and 75% *F*, respectively. For ADI treatments, a drip irrigation pipe was laid at each of the both ends per plot with a 40 cm distance from tomato plants roots. Only one of the two drip irrigation pipes in each plot was used at each irrigation time; the two drip irrigation pipes were turned on alternately so that only one side of the planting plot was irrigated with water at a time. Each irrigation treatment was repeated three times giving a total of 12 planting plots. The high-pressure low-density polyethylene film (Jingjiang Xinfeng Plastic Factory, Jiangsu, China) covered on each plot surface. The film was transparent and 0.014 mm thick. The embedded inner inlay flat drip irrigation pipes (Dayu Water-saving Co. Ltd., City, China) were 16 mm in diameter, 0.3 mm in thickness and 30 cm in emitter spacing, with operating pressure of 0.1 MPa and drip flow rate of 1.2 L/h.

Soil moisture content (SMC) was measured and controlled using Field TDR 200 probes (manufactured by Spectrum, USA). A probe was installed into 100 cm depth of the soil at the center of each plot. SMCs were measured at intervals of 10 cm down to 60 cm in depth. And, the oven-drying method was used to further calibrate the soil moisture value. Soil water was supplemented when its value reached the low limits, and the irrigation amount was calculated as following:

$$M=s\rho_{b}ph\theta_{f}\left(q_{1}-q_{2}\right)/\eta .$$

where *M* is irrigation amount, m^3^; *s* is the planned wetting area, m^2^; $\rho_{b}$ is the soil bulk density of 1.35 g·m^−3^; *p* is the wetting ratio, 0.8; h is the wet layer depth with the value, 0.4 m; $\theta_{f}$ is the maximum field capacity, 31.54%; *q*_1_ and *q*_2_ are the upper limits of the irrigation and measured SMC, respectively, %*F*; *η* is water use coefficient, *η* = 0.95.

### 2.2. Measurements

#### 2.2.1. Plant Height and Stem Diameter

After tomatoes transplant, three plants were randomly selected and marked in each plot, eliminating the marginal plants. The height of marked plants was measured using ruler with the accuracy of 1 mm, during the period from planting to pruning. The stem diameter was measured using vernier caliper by cross method per every 10 d after planting, and the measured position was at the third internode of the plants base.

#### 2.2.2. Net Photosynthetic Rate, Leaf Area Index, and Photosynthetic Pigment

The leaf net photosynthetic rate was determined using portable photosynthetic apparatus LI-6400 (manufactured by LI-COR, Lincoln, NE, USA) in Flowering period (FP), Full fruit period I (FFP1), Full fruit period II (FFP2), and Mature period (MP), and the measurement time was from nine to eleven in morning. FP, FFP1, FFP2, and MP were 0–50 d, 50–100 d, 100–140 d, and 140–180 d after tomato planting in field, respectively. The LI-6400 was operated under the conditions of an open air circuit, a built-in light source and a light intensity of 800 μmol·m^−2^·s^−1^. In each measurement, three plants per plot were randomly selected. Three leaves per plant, with same position in plant and fully exposure to the light were chosen to measure the leaf net photosynthetic rate. Subsequently, leaf area index was measured using a hand-held leaf area meter LI-3100C (manufactured by LI-COR, Lincoln, NE, USA).

After the net photosynthetic rate measurement completed, the selected leaves were collected and brought into laboratory for leaf photosynthetic pigment determination. Leaf photosynthetic pigments were extracted using acetone extract. The absorbance values of chlorophyll a, chlorophyll b, and carotenoids were measured by spectrophotometer colorimetric method at 665 nm, 665 nm and 470 nm, respectively. Total chlorophyll = chlorophyll a + chlorophyll b, the unit is mg of Total chlorophyll per g of leaves; Chlorophyll a/b = chlorophyll a /chlorophyll b.

#### 2.2.3. Analysis of Dry Matter and Roots

At beginning of the mature period, three plants were randomly selected and marked per plot. The above parts of pre-marked plants were cut off, collected and numbered after their fruits harvest. Fresh plants were weighed and dried in the oven of drum wind drying (105 ℃ for 30 min, then 75 ℃ for 36 h) and next dry plants were weighed.

The root samples of pre-marked plants were collected using the whole excavation method after removing the above parts. Soil and roots were excavated from an area of 40-cm × 30-cm to the maximum rooting depth (50 cm). Firstly, the root samples were taken out; secondly, the soils adherent to the root system were shaken and removed on the sterile filter paper, then put into sterile plastic tubes for soil bacterial community and nutrient analysis; Thirdly, root samples were brought into laboratory, soaked in water and rinsed to separate soil completely. Fine roots were collected from three layers of gauze cloth laid at the bottom of the flushing sink. The clean roots were placed in a Ziploc bag using tweezers and scanned by a double-sided scanner Epson Expression 1600 pro^®^ (Model EU-3, Nagano-ken, Japan), and the total root length (cm), root surface area (cm^2^), root volume (cm^3^) and number of root forks were analyzed using WinRHIZO image analysis system (WinRHIZO Pro2004b, 5.0, Lethbridge, Alberta, Canada). Next, the roots samples were dried and weighed. A portion of the root sample was subjected to root activity determination using the TTC (2, 3, 5-triphenyltetrazolium chloride)^®^ method [22].

#### 2.2.4. Tomato Yields, Quality, and Nutrient

Tomato fruits were harvested in each plot. The yields were calculated with unit of t·hm^−2^ (tons of tomato fruits per acre). The fruit soluble sugar contents were measured by the sulfuric acid-anthrone colorimetric method [23]; organic acid contents were determined using the acid-base titration method; vitamin C contents were measured by molybdenum blue colorimetry [24]; soluble protein contents were determined using the comas-G250 ^®^ staining method [25]; lycopene contents were measured by the UV-visible spectrophotometer EV300PC ^®^ (Thermo Fisher, Waltham, MA., USA); and the sugar to acid ratio was calculated as soluble sugar content divided by organic acid content.

The total nitrogen contents of soil samples adherent to the root (collected as in 2.2.3) and tomato fruits were determined by Semi-trace kjeldahl method [26]; the total phosphorus contents were measured by sulfuric acid–perchloric acid molybdenum antimony digests using the colorimetric method [27], and the organic carbon contents were determined by the potassium dichromate titration method [28].

#### 2.2.5. Soil Bacteria Sequence and Diversity

The soil samples collected in 2.2.3 were analyzed the bacteria sequence and diversity using high-throughput sequencing method [29].

### 2.3. Data Analysis

Data were analyzed using SPSS version 22.0 software (New York, NY, USA). The normality and homogeneity of the data for each variable were tested before the statistical analyses. And, the one-way ANOVA (analysis of variance using Duncan method) and correlation analysis were performed. Tables and plots were drawn in Excel 2010 (Redmond, WA, USA).

## 3. Results

### 3.1. Tomato Growth

#### 3.1.1. Plant Height, Stem Diameter, and Leaf Area Index

**Figure 1:**
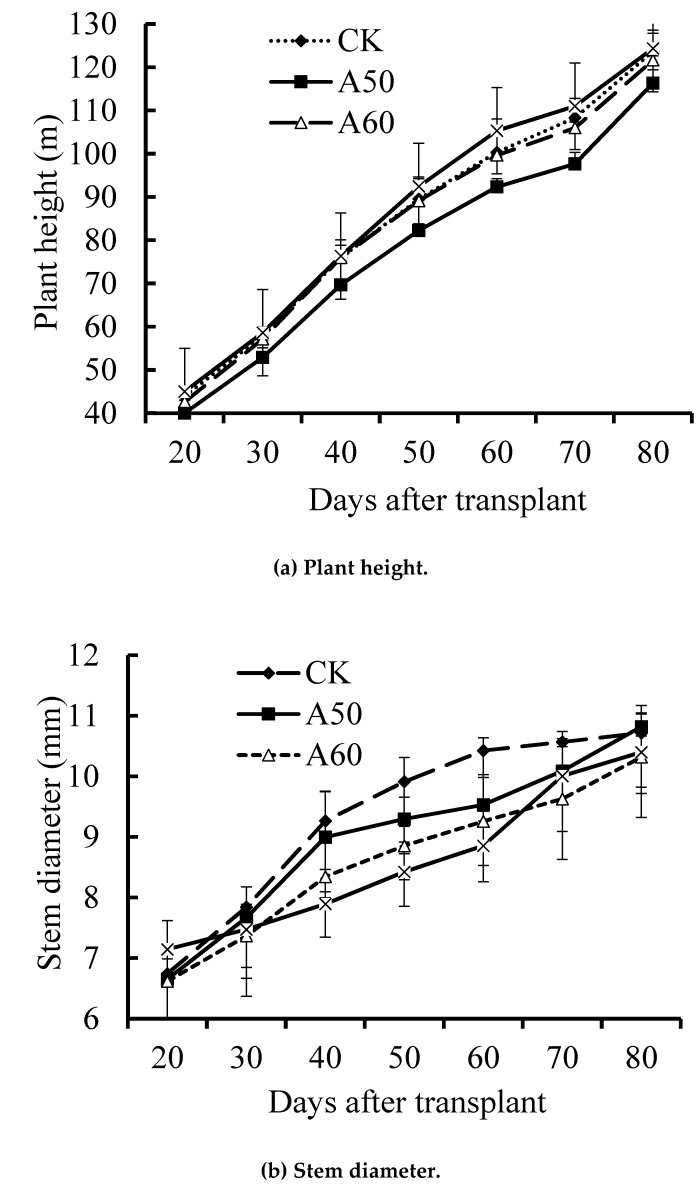
Plant height and stem diameter. CK = control.

The growth rate of plant stem was the ratio of the net growth value between the adjacent measurements to the previous measurement value, with the previous value as 100%. The results showed significant differences between the four treatments during some growth stages (Figure 1b). For example, from 20 d to 40 d after planting, the stem growth rates of A50, A60, and A70 (38%, 35%, and 26%) were 3.56, 3.28, and 2.44 times that in CK (10.67%) (*p* < 0.05), respectively; similarly, from 60 d to 80 d, the stem growth rate of A50, A60, and A70 (13.33%, 11.67%, and 17.67%) showed 4.99, 4.37, and 6.62 times that in CK (2.67%), respectively. However, the height growth rate (defined as the stem growth rate) resulted no significant difference among four treatments (Figure 1a).

**Figure 2:**
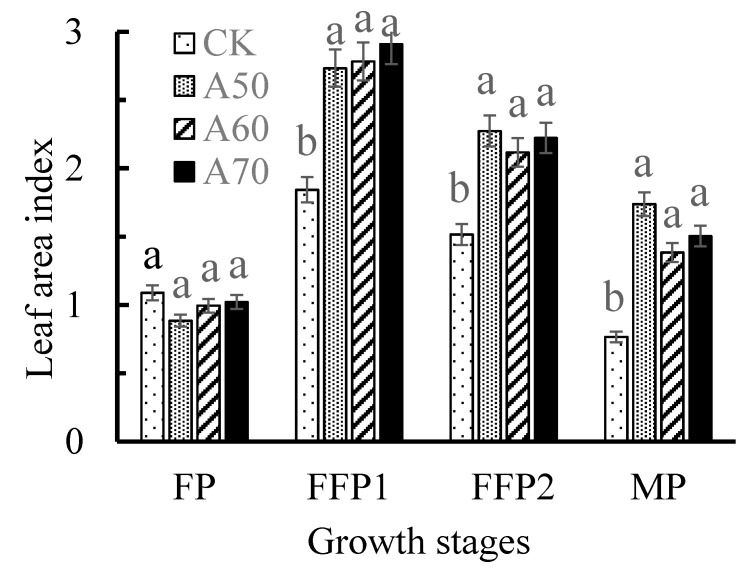
Leaf area index at different growth stages under alternate partial root-zone drip irrigation. Note: Different small letters mean significant difference (*p* < 0.05) among the different treatments in same growth stage according to Duncan test. Flowering period (FP) was 0–50 d after tomato planting to field, Full fruit period I (FFP1) was 50–100 d after tomato planting to field, Full fruit period II (FFP2) was 100–140 d after tomato planting to field, and Mature period (MP) was 140–180 d after tomato planting to field.

As found in Figure 2, the leaf area indexes value of A50, A60, and A70 were 1.48, 1.51, and 1.58 times that of CK in FFP1, respectively; showed 1.49, 1.39, and 1.46 times that of CK in FFP2, respectively; and resulted 2.25, 1.79, and 1.96 times that of CK in MP, respectively.

#### 3.1.2. Photosynthetic Pigment Contents and Net Photosynthetic Rate (Pn)

**Figure 3:**
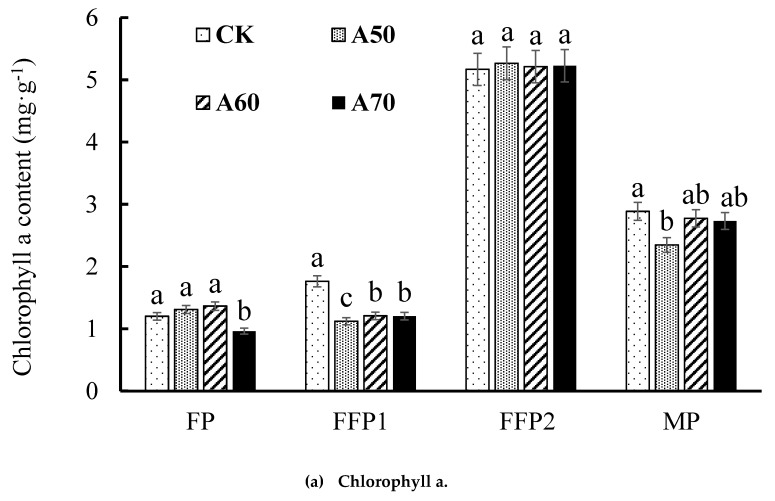

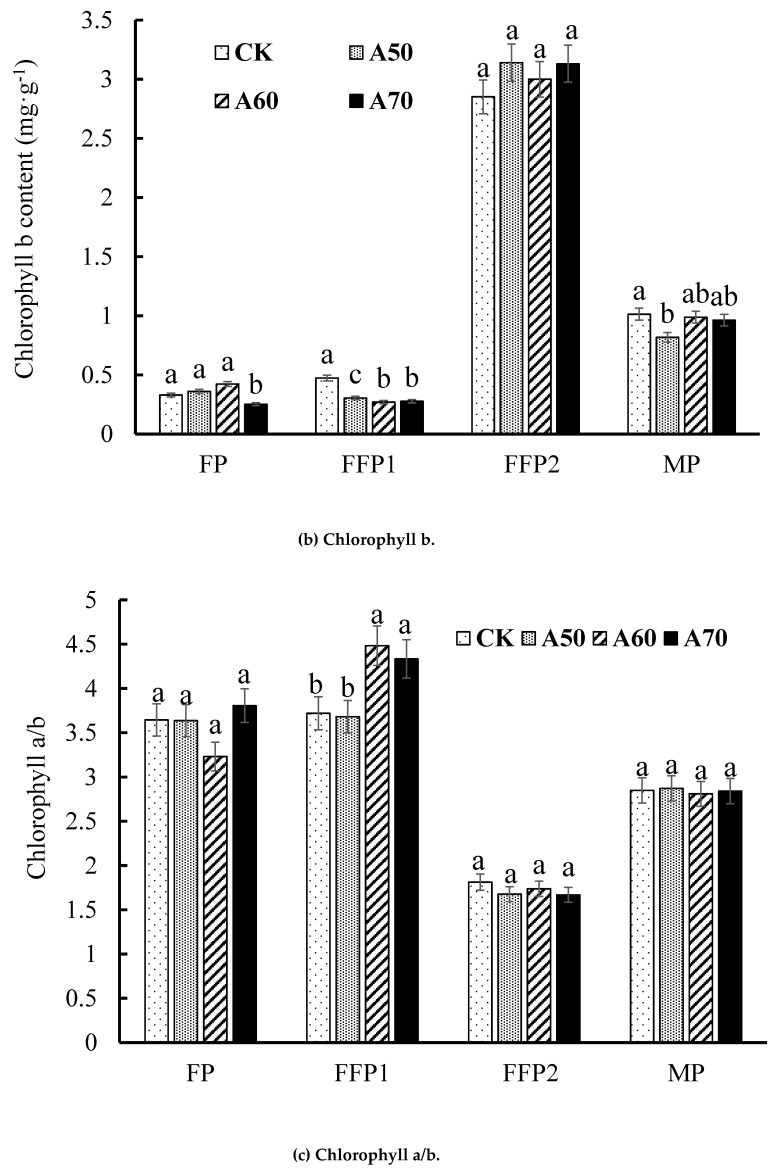

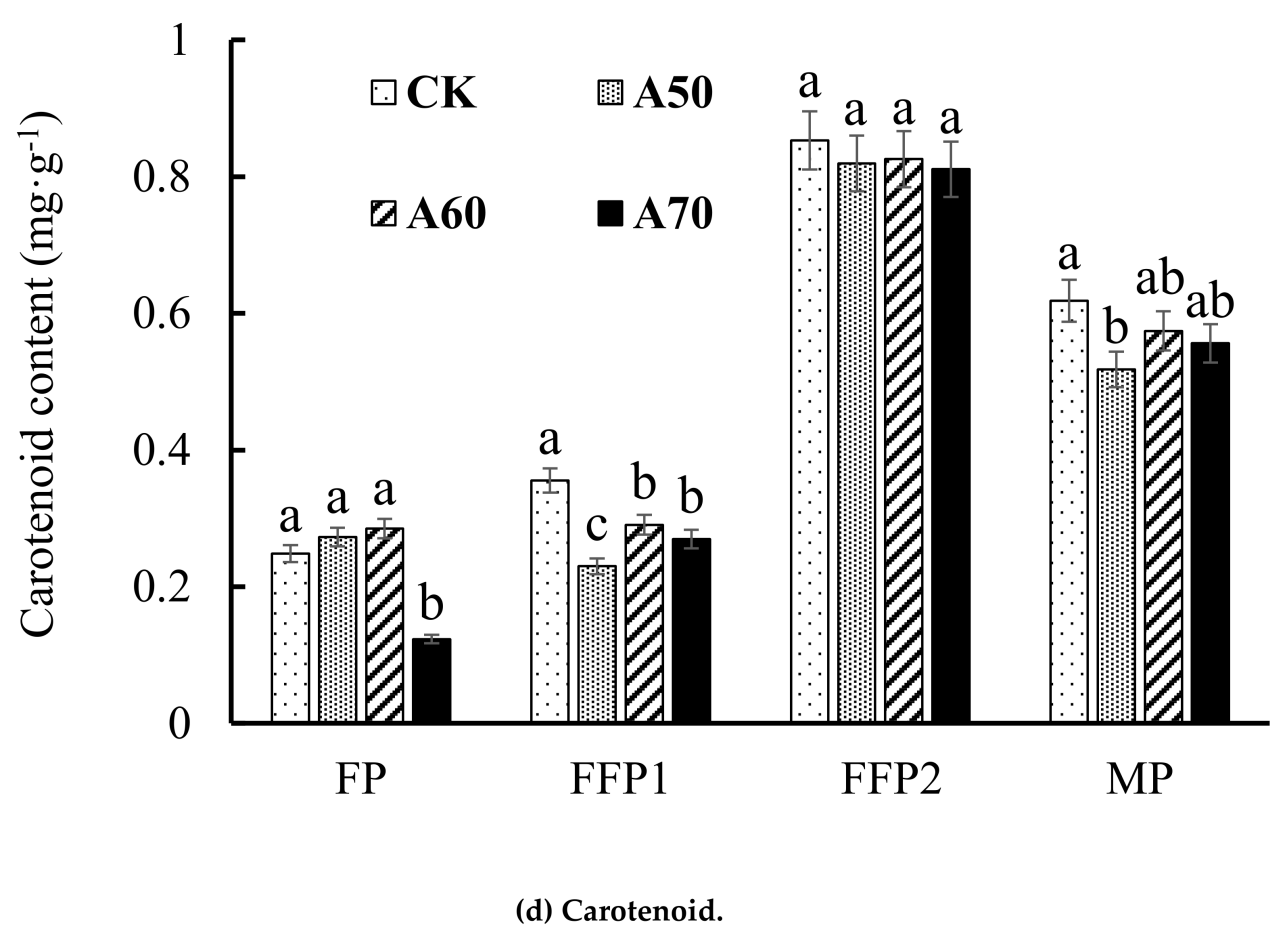
Photosynthetic pigment content in leaf at different growth stages under alternate drip irrigation (ADI). Note: Different small letters mean significant difference (*p* < 0.05) among the different treatments in same growth stage according to Duncan test. FP was 0–50 d after tomato planting to field, FFP1 was 50–100 d after tomato planting to field, FFP2 was 100–140 d after tomato planting to field, and MP was 140–180 d after tomato planting to field.

The contents of chlorophyll a, chlorophyll b, and carotenoid in CK at FFP1 stage were significantly increased than those in ADI; however, the value of Chlorophyll a/b was significantly decreased by 20.49% and 16.71% than that in A60 and A70 (Figure 3).

The *Pn* of A60 in FFP1 significantly increased 18.80% more than that in CK, but in FFP2 and MP, decreased 11.91% and 9.18% compared to those in CK; the *Pn* of A70 in FP and FFP1 increased 29.11% and 27.74% more than those in CK; however, in MP, this reduced 8.6% compared to that in CK (Table 1).

#### 3.1.3. Root Growth

**Figure 4:**
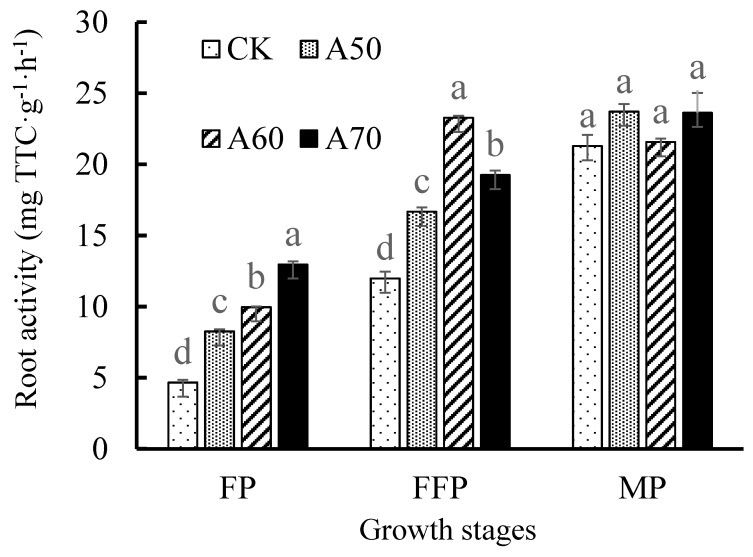
Tomato root activity at different growth stages under ADI. Note: Different small letters mean significant difference (*p* < 0.05) among the different treatments in same growth stage according to Duncan test. FP was 0–50 d after tomato planting to field, FFP1 was 50–100 d after tomato planting to field, FFP2 was 100–140 d after tomato planting to field, and MP was 140–180 d after tomato planting to field.

Tomato root length, area and forks under ADI were greatly improved (Table 2). Root length of A50, A60, and A70 showed 1.71, 1.41, and 1.27 times that of CK, respectively; root area increased by 44.87%, 33.05%, and 28.96% than that in CK, respectively; and root forks were 2.60, 2.26, and 2.86 times that of CK, respectively. Additionally, the root length indicated a decrease trend along with irrigation lower limit increase under ADI. And, the root volume of A50 and A70 were 1.42 and 1.36 times of that in CK. Moreover, root activity of the four treatments exhibited significant difference in FP and FFP (Figure 4). Root activity of A50, A60, and A70 in FP were 1.77, 2.13, and 2.78 times that of CK. Similarly, root activity of A50, A60, and A70 in FFP were 1.39, 1.94, and 1.61 times that in CK.

#### 3.1.4. Dry Matter Accumulation

**Figure 5:**
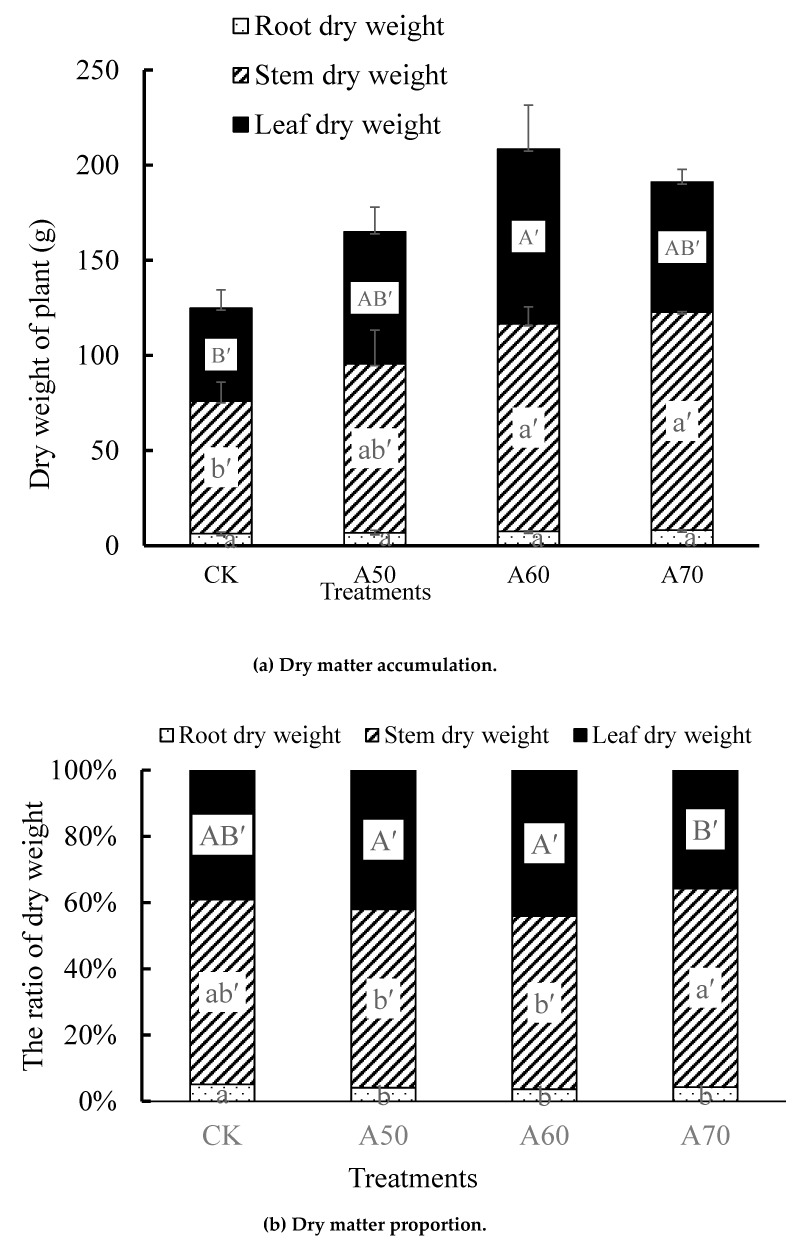
Tomato dry matter accumulation and dry matter proportion in parts of plant under ADI. Note: a(b,c) means significant difference (*p* < 0.05) among the root dry weight of different treatments; a’(b’,c’) means significant difference (*p* < 0.05) among the stem dry weight of different treatments; A’(B’,C’), means significant difference (*p* < 0.05) among the leaf dry weight of different treatments. The difference is according to Duncan test.

The total dry matter of A60 and 70 showed 1.67 and 1.53 times that of CK (Figure 5a). Although the root dry matter had no significant difference between the four treatments, the stem and leaf dry matter showed significant differences. The stem dry matter of A60 and A70 were 1.56 and 1.64 times that of CK, respectively; and the leaf dry matter of A60 was 1.88 times that of CK, respectively.

The proportion of root dry matter to total dry matter in CK was significantly higher than that in ADI. Compared with A50 and A60, A70 improved the proportion of stem dry matter in total dry matter (Figure 5b).

### 3.2. Tomato Fruits Quality, Yield, and IWUE

A50 increased soluble sugar, total soluble solid and lycopene by 38.08%, 19.48% and 30.05% than that of CK (Table 3), respectively. A60 improved soluble sugar, total soluble solid, soluble protein, lycopene, and sugar-acid ratio to be 2.06, 1.26, 1.61, 1.4, and 3.2 times that in CK, respectively. A70 decreased organic acid 43.75% than that in CK and improved sugar-acid ratio 1.97 times that of CK.

As reported in Table 4, A70 improved yields by 24.6% compared to CK. Irrigation amount of A50 decreased by 29.86% in comparison with CK. *IWUE* under A50, A60, and A70 were enhanced by 45.79%, 19.54%, and 17.05% compared to CK, respectively.

## 4. Discussion

### 4.1. Photosynthetic Efficiency

In this study, the smaller and moderate lower irrigation limits (50% and 60% field capacity) under ADI could not improve tomato leaf photosynthetic efficiency; however, when lower irrigation limit reached at 70% field capacity, ADI significantly increased tomato leaf photosynthetic efficiency in FP and FFP1 and was not significantly different from CDI (surface drip irrigation, 70% field capacity) in FFP2. Therefore, under the same irrigation condition, ADI increased tomato leaf photosynthetic efficiency. Leaf chlorophyll plays an important role on the light energy absorption, transmission and conversion in plant photosynthesis [30], that directly affects the photosynthetic efficiency and biomass accumulation. Compared with CDI, ADI failed to improve chlorophyll a and chlorophyll b content; however, chlorophyll a/b values in FP1 under ADI of 60% and 70% field capacity were significantly enhanced compared to CDI. Chlorophyll a and chlorophyll b had different absorption spectra, and the maximum absorption spectra were 420–663 nm and 460–645 nm, respectively. And, only a few chlorophyll a in the excited state could convert light energy into electrical energy [31]. As a result, appropriately increased chlorophyll a/b value can strengthen the rate of light energy utilization by leaf and enhance photosynthesis [32].

Previous experiments showed that ADI significantly increased leaf chlorophyll contents [33], which is different from the results in this study. The reason may be that they carried out the pot experiment and applied additional fertilizer. The other studies indicated that compared with CDI, ADI slightly reduced the leaf photosynthetic pigment content and net photosynthetic rate [10,13], and this is consistent with the partly results in this study. However, these studies did not analyze the effects of ADI on leaf area index. Leaf area index is significantly correlated with the whole actual photosynthetic efficiency and primary photosynthetic productivity of plant [34]. Further, in this study, ADI significantly increased the leaf area index in FFP and MP, and thus actually improved the tomato photosynthesis efficiency.

### 4.2. ADI with Excessive Drought Stress Limited Tomato Growth

Plant growth is subject to root system. Under same irrigation water amount, ADI increased tomato root length, area and volume compared to CDI [35], which is in agreement with the results of this study (Table 2), but they did not analyze the root activity. However, root activity is an important index for representing the capacity of soil moisture and nutrient absorbed by root system [36,37]. Our measurements showed that tomato root activity in the period of flowering and full fruit were increased by ADI (Figure 4). Deeply, the leaf area index in flowering and full fruit period were significantly correlated with root activity (Table 5). In addition, root area and forks were also increased by ADI and leaf area index in mature period had a significant relation to root area and forks (Table 5). Therefore, the “root-plant” interaction was enhanced by ADI. The measurements also found that diversity of soil bacterial community was significantly correlated with root length and area (Table 5), and similarly, the “root-soil” interaction was also increased [38]. Many scientific experiments and production practice proved that increase of the “root-soil-plant” interaction could enhance soil nutrient availability and uptake [39]; however, this is only partly consistent with our experimental results (Table 6). Our measured indicators revealed that the excessive drought stress (50% field capacity) weakened soil nitrogen and phosphorus utilization in ADI condition and limited soil nitrogen absorbed by tomato roots and stem; nevertheless, soil nitrogen and phosphorus absorption by the root system, plants and fruit were significantly promoted when irrigation water increased (60% and 70% field capacity). However, ADI markedly improved tomato stem diameter growth rate in 20–40 d and 60–80 d after planting (Figure 1b), and this is conducive to transportation and exchange of soil moisture, nutrients and photosynthetic product between plants and roots, and can increase photosynthesis and biomass accumulation [40]. Therefore, ADI with 60% and 70% field capacity improved tomato plant growth in a whole level through promoting soil nitrogen and phosphorus absorption by tomato and partly increasing tomato stem diameter growth rate (Figure 5a).

### 4.3. ADI Optimized Photosynthetic Products Distribution and Enhanced Tomato Yield

ADI improved tomato photosynthesis efficiency in a whole level and promoted “root-soil-plan” interaction, and this would definitely affect tomato biomass accumulation and production [41,42]. Tomato biomass and yield are related to photosynthesis and root nutrition, as well as photosynthetic products allocation [43]. In this study, ADI reduced the root dry matter proportion in total dry matter (Figure 5). However, ADI was also been found to improve tomato root dry matter in greenhouse pot experiment [44], and this is inconsistent with our results. This may depend on the environmental differences in root-zone soil created by pot and field conditions. Further, only appropriate, but not excessive, ratio of root dry matter in total dry matter can promote moisture and nutrient absorption by plants [45,46], and it will be more conducive to distribute more photosynthate into the above-ground parts of plant [47,48], when root growth indexes (length, area, and forks) and root activity were increased significantly. Fruit yield was associated with increased plant size (stem diameter) due to increased translocation of nutrients in the large stems [49]. Here, we observed stem dry matter increasing would improve tomato yield (Table 7). Although 50% and 60% field capacity ADI increased total dry matter compared to CDI and decreased the proportion of root dry matter to total dry matter, they decreased the ratio of stem dry matter in total dry matter (Figure 5b). Therefore, this might lead to the tomato production of 50% and 60% field capacity ADI were not significantly higher than that of CDI.

ADI of 70% field capacity not only decreased the proportion of root dry matter in total dry matter but also enhanced stem dry matter proportion in total dry matter, and this resulted significantly higher yield than CDI. Additionally, the irrigation amounts of 50% and 60% field capacity ADI were 10–20% lower than CDI of 70% field capacity, and the relative deficit in soil moisture could lead to less photosynthetic distributed into fruits [50]. When the field capacity was 70%, the water deficit stress decreased, and ADI was conducive to photosynthetic distributed into fruit distribution and increased tomato production.

### 4.4. Fruit Quality and IWUE

With regard to the effect of drip irrigation on tomato quality, the existing studies have paid more attention to the regulation function of drip irrigation and fertilizer coupling on individual quality indicators of tomato, such as soluble solids, organic acid, vitamin C, and other individual indicators [1]. There are less studies on comprehensive evaluation on the response of tomato quality indexes to drip irrigation. The regulation mechanism of drip irrigation on tomato quality is still not clear. Moreover, there is contradiction in the existing research results. Some studies suggested that soil moisture deficit under drip irrigation could improve tomato quality [1], the other studies have found that drip irrigation increased tomato yield but significantly reduced its quality, such as SSC (soluble solids content) and lycopene [51], vitamin C, organic acids, and pH value [52].

**Figure 6:**
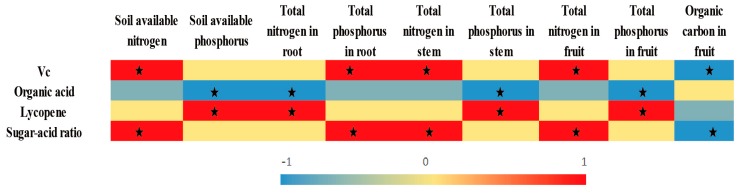
Correlation analysis between tomato fruit quality and soil and plant nutrient content. Note: Different color depth mean correlation difference, correlation value is showed by the value of number from −1 to 1, ★ means significance correlation (*p* < 0.05) correlation.

In order to response to the above questions, we analyzed the responses of seven common indicators of tomato quality to soil moisture deficit under ADI. The results revealed that ADI with moderate soil moisture deficit (60% field capacity) was best beneficial to significantly improve tomato quality index, and ADI with the larger or light soil moisture deficit (50% or 70% field capacity) was the second beneficial to tomato fruit quality. Soil moisture deficit could affect soil nutrient activity and absorption, as well as photosynthesis product distribution [43]. The correlation between tomato quality and soil nutrients activity and absorption (Figure 6) revealed that, under ADI conditions, tomato fruits vitamin C and sugar-acid ratio were significantly positively correlated with soil nitrogen effective, phosphorus content in root, nitrogen content in stem and fruit; lycopene was significantly related with soil available phosphorous, nitrogen content in root, phosphorus content in stem, and phosphorus content in fruit. Therefore, appropriate soil moisture deficit might improve tomato fruit quality by enhancing soil nitrogen and phosphorus activity, promoting soil nitrogen and phosphorus absorption and improving nitrogen and phosphorus content in fruit (Table 6). However, 60% field capacity significantly improved tomato quality index by five out of seven, while 70% field capacity increased only two-sevenths quality indexes. The reason might be the increased soil moisture [53], but the deeper causes still need to be studied. And, 50% field capacity could create excessive drought stress and lead to tomato fruit quality inferior to 60% field capacity. ADI was found to improve soluble sugar content and reduced organic acids content, thus improving acid-sugar ratio in some studies [54]. That is consistent with the results of this study. However, their results also showed ADI significantly increased the content of tomato vitamin C, which is not accordance with the results in this study. The reason may be that the calcium fertilizer was applied in those studies, and calcium fertilizer could promote photosynthesis, thus being conducive to vitamin C synthesizing [55]. And, the soluble solids content of tomato fruits was reduced with soil moisture increase under drip irrigation [53], whereas we found that soluble solids showed a trend of increasing then decreasing along with soil moisture increase of ADI and reached the maximum in medium soil moisture. This difference is due to the use of sandy loam in that study. In addition, these previously mentioned studies on tomato quality all did not further analyze the relationship between tomato quality and soil nutrient activation and utilization.

In this study, ADI not only significantly promoted tomato growth but also improved *IWUE*. ADI of 50% field capacity increased *IWUE* 45.79% than that of CDI; ADI of 60% and 70% field capacity had no significant difference with CDI in water consumption but increased *IWUE* 19.54% and 17.05% more than CDI. Therefore, comprehensive consideration on tomato growth, yield, fruit quality, and *IWUE,* ADI of 60% or 70% field capacity would be recommended for tomato production.

## 5. Conclusions

ADI increased tomato root length, area, and forks and root activity, and it enhanced the photosynthesis by improving leaf area index in full fruit period and mature period. Moreover, leaf area index in mature period was significantly correlated with root area and forks, and soil bacterial community diversity showed a significant correlation with root length and area. These positive factors increased “root–soil–plant” interaction and were beneficial for improving soil nitrogen and phosphorus availability and absorption by tomato plants and promoting their growth. However, soil moisture deficit significantly affected tomato photosynthate accumulation and distribution and fruit quality. Compared with CDI, ADI of 70% field capacity significantly promoted photosynthetic transfer into fruits and moderately improved fruits quality, increased yield by 24.6%, and enhanced *IWUE* by 17.05%; ADI of 60% field capacity significantly promoted *IWUE* by 19.54%, greatly improving fruits quality and did not reduce yield.

## Figures and Tables

**Table 1:** Tomato net photosynthetic rate at different growth stages under ADI.

Treatments	Net Photosynthetic Rate *Pn* (μmol·m^−2^·s^−1^)			
	FP	FFP1	FFP2	MP
CK	8.9 b	10.8c	13.9a	12.2a
A50	9.7b	11.4c	13.9a	12.1a
A60	9.8b	12.8b	12.2b	11.0b
A70	11.5a	13.8a	15.4a	11.2b

Note: FP, FFP1, FFP2, and MP were flowering period, full fruit period Ⅰ, full fruit period II, and mature period, respectively. Different letters mean significant difference (*p* < 0.05) among the different treatments according to Duncan test.

**Table 2:** Root growth characteristics in MP.

Treatments	Root Length (cm)	Root Area (cm^2^)	Number of Root Forks	Root Volume (cm^3^)
CK	1473.7d	721.0b	3969c	29.0b
A50	2524.9a	1044.6a	10307ab	41.1a
A60	2080.0b	959.3a	8979b	37.5ab
A70	1869.3c	929.8a	11371a	39.2a

Note: Different letters mean significant difference (*p* < 0.05) among the different treatments according to Duncan test.

**Table 3:** Fruit quality under ADI.

Treatments	Soluble Sugar (%)	Total Soluble Solid (%)	Soluble Protein (Mg·g^−1^)	Vc (Mg·100 g^−1^)	Organic Acid (%)	Lycopene (Μg·g^−1^)	Sugar-Acid Ratio
CK	2.60c	5.80c	2.69b	15.76ab	0.32a	61.00c	8.13c
A50	3.59b	6.93b	2.83b	14.55b	0.29ab	79.33b	12.41bc
A60	5.35a	7.30a	4.32a	18.20a	0.21ab	85.38ab	26.04a
A70	2.60c	6.03c	2.57b	15.32ab	0.18b	90.00a	16.00b

Note: Different letters mean significant difference (*p* < 0.05) among the different treatments according to Duncan test.

**Table 4:** Yield, irrigation amount, and irrigation water use efficiency (IWUE) under ADI.

Treatments	Yield (t· hm^−2^)	Irrigation Amount (mm)	IWUE (kg·m^−3^)
CK	75.21c	291.17ab	44.16c
A50	77.13b	204.78c	64.38a
A60	84.75ab	274.42b	52.79b
A70	93.71a	309.89a	51.69b

Note: Different letters mean significant difference (*p* < 0.05) among the different treatments according to Duncan test.

**Table 5:** Correlation analysis among plant root, plant growth indexes, and soil bacteria.

	Leaf Area Index in FP	Leaf Area Index in FFP	Leaf Area Index in MP	Soil Bacteria Sequence	Soil Bacteria Diversity
Root activity in FP	0.317	-----	-----	-----	-----
Root length in FP	−0.634	-----	-----	-----	-----
Root area in FP	−0.648	-----	-----	-----	-----
Root volume in FP	−0.864	-----	-----	-----	-----
Root forks in FP	−0.702	-----	-----	-----	-----
Root activity in FFP	-----	0.974 *	-----	-----	-----
Root length in FFP	-----	−0.133	-----	-----	-----
Root area in FFP	-----	0.174	-----	-----	-----
Root volume in FFP	-----	0.543	-----	-----	-----
Root forks in FFP	-----	−0.743	-----	-----	-----
Root activity in MP	-----	-----	0.816	0.998 *	0.267
Root length in MP	-----	-----	0.852	0.182	0.988 *
Root area in MP	-----	-----	0.962 *	0.250	0.996 *
Root volume in MP	-----	-----	0.967 **	0.903	0.610
Root forks in MP	-----	-----	0.970*	0.905	−0.225

**. Correlation is significant at the 0.01 level; *. Correlation is significant at the 0.05 level (2-tailed).

**Table 6:** Soil nutrient and tomato growth indexes.

**Treatments**	**Soil Available Nitrogen**	**Soil Available Phosphorus**	**Total Nitrogen in Root (%)**	**Total Phosphorus in Root (%)**	**Total Nitrogen in Stem (%)**
CK	46.91d	94.43c	1.68c	0.19c	1.58c
A50	69.27c	140.75bc	1.67c	0.19b	1.38d
A60	102.55a	155.71b	1.80b	0.36a	1.91a
A70	89.61a	274.54a	1.92a	0.29a	1.77b
	**Total Phosphorus in Stem (%)**	**Total Nitrogen in Fruit (%)**	**Total Phosphorus in Fruit (%)**	**Organic Carbon in Fruit (%)**	
CK	0.089d	2.22c	0.37c	39.58a	
A50	0.092c	1.81d	0.35d	38.55b	
A60	0.156b	2.90a	0.51b	39.39a	
A70	0.253a	2.83b	0.53a	39.20a	

Note: Different letters mean significant difference (*p* < 0.05) among the different treatments according to Duncan test.

**Table 7:** Correlation analysis between tomato yield and dry matter.

	Yield	Leaf Dry Weight	Stem Dry Weight	Root Dry Weight	Total Dry Weight
Yield	1	0.279	0.605 *	0.441	0.472
Leaf dry weight		1	0.765 **	0.616 *	0.936 **
Stem dry weight			1	0.924 **	0.943 **
Root dry weight				1	0.827 **
Total dry weight					1

**. Correlation is significant at the 0.01 level; *. Correlation is significant at the 0.05 level (2-tailed).
